# Unraveling the Impact of COVID-19 on Rheumatoid Arthritis: Insights from Two Romanian Hospitals—Preliminary Results

**DOI:** 10.3390/biomedicines12092145

**Published:** 2024-09-21

**Authors:** Andreea-Iulia Vlădulescu-Trandafir, Gelu Onose, Constantin Munteanu, Ioana Iancu, Andra-Rodica Bălănescu, Daniela Opriș-Belinski, Florian Berghea, Cristiana Prefac, Elena Grădinaru, Sorina Aurelian, Vlad Ciobanu, Violeta-Claudia Bojincă

**Affiliations:** 1 Faculty of Medicine, University of Medicine and Pharmacy “Carol Davila”, 020022 Bucharest, Romania; gelu.onose@umfcd.ro (G.O.); andra.balanescu@umfcd.ro (A.-R.B.); daniela.opris@umfcd.ro (D.O.-B.); florian.berghea@umfcd.ro (F.B.); sorina.aurelian@umfcd.ro (S.A.); violeta.bojinca@umfcd.ro (V.-C.B.); 2 Neuromuscular Rehabilitation Clinic Division, Teaching Emergency Hospital “Bagdasar-Arseni”, 041915 Bucharest, Romania; 3 Faculty of Medical Bioengineering, University of Medicine and Pharmacy “Grigore T. Popa” Iasi, 700454 Iasi, Romania; 4 Manchester Centre for Clinical Neuroscience, Manchester M6 8HD, UK; ioana.iancu1811@gmail.com; 5 Department of Internal Medicine and Rheumatology, “Sfânta Maria” Hospital, 011172 Bucharest, Romania; cristiana.prefac@gmail.com (C.P.); elena.gradinaru@rez.umfcd.ro (E.G.); 6 Gerontology and Geriatrics Clinic Division, St. Luca Hospital for Chronic Illnesses, 041915 Bucharest, Romania; 7 Computer Science Department, Politehnica University of Bucharest, 060042 Bucharest, Romania; vlad.ciobanu@upb.ro

**Keywords:** rheumatoid arthritis, COVID-19, clinical characteristics, treatment outcomes, severity

## Abstract

Background: Rheumatoid arthritis (RA) patients are at heightened risk of Coronavirus Disease—19 (COVID-19) complications due to immune dysregulation, chronic inflammation, and treatment with immunosuppressive therapies. This study aims to characterize the clinical and laboratory parameters of RA patients diagnosed with COVID-19, identify predictive risk factors for severe forms of this infection for RA patients, and determine if any RA immunosuppressive therapy is associated with worse COVID-19 outcomes. Methods: A retrospective observational case-control study included 86 cases (43 diagnosed with RA and 43 cases without any inflammatory or autoimmune disease) that suffered from SARS-CoV-2 in two Romanian hospitals between March 2020 and February 2024. Data on demographics, RA disease characteristics, COVID-19 severity, treatment regimens, and outcomes were analyzed. Results: RA patients exhibited a distinct symptom profile compared to non-RA controls, with higher incidences of neurological, musculoskeletal, and gastrointestinal symptoms, while the control group showed more respiratory and systemic manifestations. Severe COVID-19 is correlated with age and laboratory markers like erythrocyte sedimentation rate (ESR), leucocytes, neutrophils, neutrophil-to-lymphocyte ratio (NLR), aspartate aminotransferase (AST), serum creatinine, and urea. Additionally, RA treatments, particularly rituximab (RTX), were associated with more severe COVID-19 outcomes (but with no statistical significance), potentially due to the advanced disease stage and comorbidities in these patients. Post-infection, a significant number of RA patients experienced disease flares, necessitating adjustments in their treatment regimens. Conclusions: This study underscores the complex interplay between RA and COVID-19, highlighting significant clinical heterogeneity and the need for tailored management strategies. Limitations include sample size constraints, possible selection, and information bias, as well as the lack of adjustments for potential confounding variables that hinder the ability to formulate definitive conclusions. Future research plans to expand the research group size and further elucidate these relationships.

## 1. Introduction

In recent years, there has been an incremental concern in the illness called “COVID-19” or novel coronavirus disease 2019 (nCov19), as per the classification by the World Health Organization (WHO): a multisystem illness caused by a ribonucleic acid (RNA) virus from the coronavirus family referred to as “severe acute respiratory syndrome coronavirus 2” or SARS-CoV-2 [1,2,3]. The worldwide spread of COVID-19 has significantly affected the lives of people around the globe. It has brought immense challenges to public health and economies and changed how we go about our daily lives [1,4].

As of June 2024, the COVID-19 pandemic has resulted in over 700 million reported cases and 7 million fatalities. In Romania, there were approximately 3.5 million confirmed cases, with nearly 70,000 deaths, according to a report from the beginning of the same period mentioned above. Despite a downward trend in infection rates and mortality, intermittent surges in cases continue to occur, leading to ongoing daily reports of new infections and fatalities [3,5].

Despite the initial consideration of COVID-19 as primarily a respiratory ailment [2,6], subsequent research has revealed its multifaceted impact on innate and adaptive immunity. This includes the augmentation of interferon and cytokine responses, induction of endothelial activation and inflammation, and the onset of immunothrombosis. In severe cases, these mechanisms can culminate in a life-threatening cytokine storm [2]. RA is a chronic systemic inflammatory condition characterized by persistent inflammation of the synovium, resulting in joint damage, pain, and impaired functionality, influenced by genetic and environmental factors [7,8]. Considering various sources found in the literature, this disease affects between 0.24% and 1% [8,9,10] or approximately 1.2% of the adult population [1,11]. The etiology of RA involves a complex interplay of genetic predisposition, environmental triggers, and aberrant immune responses that disrupt immune tolerance. The main actors are cytokines such as tumor necrosis factor-alpha (TNF-α), interleukins (IL-1, IL-6), and other inflammatory mediators that are also found in SARS-CoV-2-infected patients in high concentrations [1,12,13].

Amid this global crisis, patients with immune-mediated inflammatory diseases (IMIDs), such as RA, represent a particularly at-risk group, necessitating focused attention and tailored management strategies. Prior studies indicate that advanced age is a significant determinant of mortality in COVID-19 cases. Furthermore, being male, having arterial hypertension, diabetes, cardiovascular (CVD) and/or kidney diseases, neoplasia, dementia, as well as treatment with high doses of corticosteroid and RTX, are all factors that increase the risk of fatality and hospitalization due to COVID-19 [1,14,15,16].

The COVID-19 pandemic has introduced additional layers of complexity and concern for RA patients. Immunosuppressive therapies, including corticosteroids and disease-modifying antirheumatic drugs (DMARDs) such as conventional synthetic (csDMARDs), targeted-synthetic (tsDMARDs), and biological (bDMARDs), are essential in managing RA. These therapies can favorably modulate but also affect the body’s immune response, thus changing susceptibility to infections, including SARS-CoV-2. From a study in our country based on the data of the Romanian Registry of Rheumatic Diseases (RRRD) in 2022, we concluded that among the 5396 RA patients, almost 90% were treated with at least one csDMARDs, nearly 80% were receiving a biologic DMARD therapy (most frequently being TNF-α inhibitors), and 21.2% were treated with tsDMARDs [1,17]. Given these findings, it is imperative to comprehensively analyze the impact of each treatment in the context of the ongoing SARS-CoV-2 infection. Furthermore, a recent study demonstrated that COVID-19 has been identified as a risk factor for poor therapeutic adherence in patients with IMIDs, including Inflammatory Bowel Disease (IBD). Studies have shown that during the pandemic, a significant number of IBD patients exhibited lower adherence to therapy, driven by fears that their treatment might increase susceptibility to SARS-CoV-2 infection. Additionally, prior COVID-19 infection was found to be a strong predictor of non-adherence, underscoring the need for targeted strategies to support these patients during such challenging times [18].

Early in the COVID-19 pandemic, it was observed that the neutrophil-to-lymphocyte ratio (NLR) was markedly higher in patients with severe or critical illness and has since been recognized as a reliable marker for assessing disease severity in COVID-19 [19,20,21]. NLR also serves as a prognostic marker in other clinical conditions, including solid tumors, COPD, sepsis, liver cirrhosis, RA, acute pancreatitis, and psoriasis [22,23].

Neutrophils play a vital role in immune system activation by releasing reactive oxygen species that can damage cellular deoxyribonucleic acid and facilitate the release of viruses from infected cells. Additionally, they promote the production of cytokines and other effector molecules, contributing to the inflammatory response. However, in cases of systemic inflammation, increased levels of IL-6 (abundant in severe COVID-19 cases) paradoxically reduce lymphocyte counts, thereby diminishing cellular immunity [19,21].

In recent years, the systemic inflammation index (SII), calculated as (neutrophil count×platelet count)/lymphocyte count, has emerged as a significant hematological marker across various medical conditions, including cancer, cardiovascular disease, and liver disease. Moreover, research has highlighted the SII’s effectiveness in distinguishing between active disease and remission in IMIDs, including RA. More recently, its utility has been explored in the context of COVID-19, where it has demonstrated a superior predictive ability for adverse clinical outcomes compared to other hematological indices [23,24,25].

Furthermore, studies from an Italian cohort identified the SII as the most critical prognostic biomarker for survival among patients infected with SARS-CoV-2 [26]. The SII measured during hospital admission has shown a strong association with disease severity and mortality in COVID-19 patients [25,27,28].

The interplay between RA and COVID-19 presents a dual challenge: managing the underlying autoimmune condition while mitigating the risk and impact of COVID-19. RA patients face a heightened risk of severe COVID-19 outcomes, compounded by the potential for flare-ups and disease exacerbation during and after infection. The burden is not only medical but also psychological and social, as these patients navigate the complexities of healthcare access, continuity of care, and the mental health impacts of prolonged isolation and stress, depression, and anxiety being incriminated as risk factors for RA flares [11,29].

The overall primary outcome of this study was to determine whether there is a discernible impact of COVID-19 on the clinical and paraclinical progression in patients with RA, comparatively with non-RA patients, based on a specific data set. The secondary outcome involved identifying predictive risk factors for severe forms of SARS-CoV-2 in RA cases in contrast with the control group. Additionally, the study aimed to determine if any RA immunosuppressive therapy is associated with worse COVID-19 outcomes.

## 2. Materials and Methods

**Ethical Considerations**: This study has the approval of the Scientific Research Ethics Committee of the “Sfânta Maria” Clinical Hospital (No. 6655/21.03.2022) and of the “Bagdasar-Arseni” Teaching Emergency Hospital (No. 17071/17.04.2024), and patient data have been coded. All patients signed an informed consent form to participate in the study.

**Study design:** We conducted a retrospective, observational case-control study on two independent groups of inpatients encompassing a total of 86 cases (43 of whom were diagnosed with RA) that suffered from COVID-19 between 1 March 2020, and 29 February 2024, in two specialized hospitals from Bucharest, Romania. The study closely adheres to the STROBE Statement, as included in Supplementary Material Table S1. 

**Inclusion and Exclusion Criteria**: In the study group, participants were comprised of individuals over 18 years of age with a confirmed diagnosis of RA according to the symptomatology and laboratory findings, alongside current classification criteria [30] and who had also tested positive for COVID-19 through reverse transcription polymerase chain reaction (RT-PCR) or rapid antigen testing. These patients were selected from the “Sfânta Maria” Clinical Hospital. The second group (the control group) included adult patients who had been diagnosed with COVID-19 using the aforementioned testing methods but who had other comorbidities aside from RA or any other immune, inflammatory, or infectious disease other than SARS-CoV-2 that could influence the laboratory tests. This group was selected from the “Bagdasar-Arseni” Teaching Emergency Hospital. Exclusion criteria included pediatric patients, uncertain COVID-19 diagnosis (based solely on symptoms), patients with uncertain (not fully confirmed: clinically and through laboratory results) RA diagnosis, a severe mental illness that could make the ability to give informed consent questionable, and refusal to provide informed consent, and specifically for the control group, any other immunological or infectious diseases. In the first phase of the study, 43 RA and 52 non-RA COVID-19 cases were included. Furthermore, after the exclusion of patients with urinary infections and pressure ulcers/sores, 43 patients remained in the control group.

**Source of Data**: Information was collected from medical records/files, the hospital’s database, and directly through face-to-face or telephone conversations, with prior informed consent obtained. Demographic data, detailed information on SARS-CoV-2 infection, and underlying RA disease were collected and analyzed descriptively. This customized, unitary clinical and paraclinical dataset included specific COVID-19 symptoms, also categorized by major organ involvement, the temporal relationship between symptoms and COVID-19 diagnosis and hospital admission, the duration of symptoms, comorbidities, and laboratory test results concerning inflammatory syndrome (including two specific indices: NLR and systemic inflammation index), the complete blood count, and biochemical markers.

**Statistical Analysis**: The initial dataset for the study was an Excel spreadsheet created using Microsoft Excel 2010. The data analysis was carried out using the International Business Machines Corporation Statistical Package for the Social Sciences version 29 (IBM SPSS Statistics for Windows, Version 29.0.2.0) [31]. For numerical variables, the data distribution was assessed using the Shapiro–Wilk test to determine the normality of the distribution in each group. If the p-value was greater than 0.05, the data were considered to follow a normal distribution. For variables with a normal distribution (*p* > 0.05 in the Shapiro–Wilk test), the independent samples t-test was used, and results were reported as mean ± standard deviation (SD). For variables that did not follow a normal distribution (*p* < 0.05 in the Shapiro–Wilk test), the non-parametric Mann–Whitney U test was applied, and results were reported as median.

The chi-square test was employed to compare categorical variables between the two groups. Proportions were reported as absolute numbers and percentages of the total group. A *p*-value of <0.05 was considered statistically significant. Furthermore, the one-way analysis of variance (ANOVA) has been employed to correlate the severity of COVID-19 and different variables. All statistical tests were deemed significant if *p* < 0.05, with a 95% confidence level and specific confidence intervals (CI) employed for calculations.

Accordingly, this study compared the clinical and paraclinical characteristics—based on specific parameters—between patients with and without RA who had experienced a SARS-CoV-2 infection, noting significant differences between the groups. However, the results should be interpreted with caution as no adjustments were made for potential confounding factors, such as age and the presence of comorbidities, because of the small sample size—and this we acknowledge as a limitation of our study—such as age and comorbidities. Hence, the observed differences may reflect not only the presence of RA but also the influence of other uncontrolled variables. Therefore, we plan to conduct adjusted analyses in the near future, utilizing increased statistical power to strengthen the foundation of our statistical data processing.

## 3. Results

### 3.1. Epidemiology; General Characteristics

Within our RA study group, 37 patients collectively experienced 43 instances of infection. Four individuals encountered reinfections, and one patient experienced two separate recrudescences. Knowing that COVID-19 tests via PCR can be positive up to 100 days in the upper respiratory tract samples [32], we reported “reinfections” as only those diagnosed one year after the initial contamination. Each infection has its particularity. Thus, we analyzed the data for the 43 infections. In the control group, a total of 43 diagnoses of SARS-CoV-2 were observed. This brings the total number of infections in our entire subject group, which includes both RA and non-RA participants, to 86. These figures were used for the statistical analysis.

**Figure 1:**
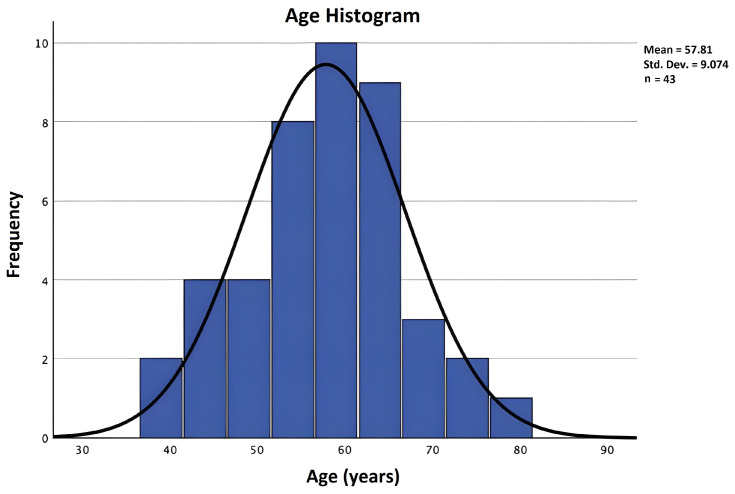
The age histogram (measured in years) for the patients in the RA sample.

In the RA research group of 43 cases, 36 were female and 7 were male. In total, 34 infections were from urban areas, while 9 were from rural areas. The median age was 57.81 ± 9.07 years, with a minimum age of 39 and a maximum age of 79. From Figure 1, we can see that the age distribution in our sample group was homogenous. Additionally, 28 cases had never smoked, 7 were current smokers, and 8 were former smokers who quit. Twenty were diagnosed through RT-PCR, and the rest through rapid antigen tests. Regarding their immunological signature, 35 had positivity for rheumatoid factor (RF) and 21 for anti-citrullinated protein antibodies (ACPAs). The Steinbrocker grading of severity of RA [33] revealed that the majority (24 cases) had stage II, 10 stage III, and 2 stage III-IV. The mean RA duration was 10.53 years. The majority (28 in the RA group and 26 in the non-RA group) were infections spread in the community without a definite infectious contact identified, underlining the extreme contagiousness of this virus.

The control group comprised 43 participants, including 17 males and 26 females, with a median age of 67.65 years (±14.65 years). Of these participants, 35 resided in urban areas, indicating a predominantly urban sample. Regarding smoking history, 7 individuals were current smokers, 35 had never smoked, and 1 was a former smoker. Diagnostic methods included RT-PCR for 37 participants, while the remaining 6 were diagnosed using rapid antigen tests.

A significant statistical disparity is obvious between the genders in the two groups (χ^2^:5.780, *p* = 0.016). Furthermore, according to the Mann–Whitney U test, there was a statistically significant difference between the two groups regarding age (z = −4.130, *p* < 0.001), with higher values in the non-RA group, as shown in Table 1, along with the demographic characteristics of both groups.

### 3.2. The Rich Tapestry of Clinical, Paraclinical, and Therapeutic Aspects of COVID-19

#### 3.2.1. Symptomatology and the Challenge of Prolonged Symptoms in COVID-19

**Figure 2:**
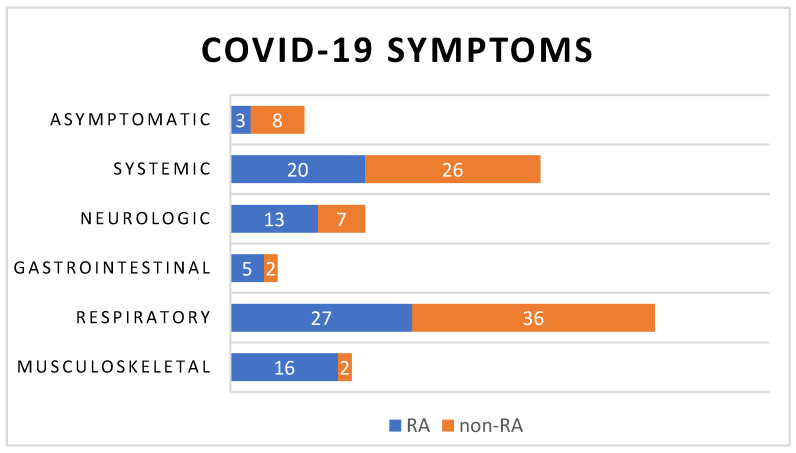
Distribution of COVID-19 clinical manifestations in the study sample.

As aforementioned, the clinical manifestation of COVID-19 encompasses a broad range of symptoms, underscoring the heterogeneous nature of the disease. Respiratory symptoms, including dyspnea, cough, rhinorrhea, and sore throat were observed in 27 cases in the RA group and 36 in the control group. Additionally, in 20 RA and 26 non-RA cases, we observed systemic symptoms, such as fever, malaise, and fatigue. Musculoskeletal symptoms manifesting as arthralgia and myalgia were predominantly in the RA group (16 cases) and only two cases in the other group. Neurological issues were identified in 13 RA and seven non-RA cases, encompassing dizziness, headaches, ageusia (loss of taste), and anosmia (loss of smell). Gastrointestinal symptoms were noted in five RA and two non-RA cases, with complaints of nausea, vomiting, stomach ache, and diarrhea. Remarkably, only three RA patients and eight non-RA patients remained asymptomatic. A significant statistical disparity is evident between the types of clinical manifestations in the two groups, as can be depicted in Table 2, using the chi-squared test. These differences can be easily observed in Figure 2.

In the observed RA cases, only five individuals reported prolonged symptoms (lasting over 28 days). These cases comprised two male and three female patients, with an average age of 61.4 years. The severity of the infections varied, encompassing one mild case, one moderate case, two severe cases, and one critical case characterized by acute respiratory distress syndrome (ARDS). The predominant symptoms observed in this specific subgroup encompassed respiratory issues such as shortness of breath, cough, and sore throat, alongside myalgias, arthralgias, malaise, and fever. Among these cases, only three necessitated hospitalization. During the infection, treatment regimens included the administration of antivirals to one patient, antibiotics to four patients, corticosteroids (specifically Dexamethasone) to two patients, and anticoagulants to three patients. Regrettably, one patient succumbed to the illness. Additionally, three patients successfully recovered from the infection but encountered enduring pulmonary complications, necessitating ongoing home-based oxygen therapy. Significantly, one of the patients necessitated a year of oxygen therapy and ultimately passed away.

The five patients had a lengthy history of RA, with an average disease duration of 11.4 years. Only one patient exhibited corticosteroid dependency. All patients were undergoing csDMARDs treatment, and four were also receiving bDMARDs. Among these, three patients were undergoing treatment with RTX, with a mean duration of 5 months since their last infusion.

Following their infection, it is noteworthy that four patients encountered disease flares within three months, necessitating a change in biological therapy. The average Disease Activity Score in 28 joints with C Reactive Protein (DAS28-CRP) was 5.17, denoting elevated disease activity. Furthermore, four patients presented with cardiovascular comorbidities, specifically arterial hypertension (HTN), while three patients had pulmonary comorbidities, encompassing pulmonary fibrosis, interstitial lung disease (ILD), asthma, and a history of pulmonary tuberculosis.

In the non-RA group, nine cases exhibited prolonged symptoms, with five of these individuals being female and having an average age of 72.33 years. The severity of the cases included six severe and three critical instances. All nine patients experienced respiratory involvement, with eight also presenting systemic symptoms and three displaying neurological symptoms such as ageusia, anosmia, and disorientation/confusion. Treatment for these patients included antibiotics, anticoagulants, and symptomatic care; eight of them also received antivirals and corticosteroids. Regarding oxygen therapy, all patients required oxygen administration via nasal cannula (NC), with flow rates ranging from 6 to 15 L per minute. Additionally, four patients required continuous positive airway pressure (CPAP) therapy, and two underwent orotracheal intubation. The outcomes were significant, with two patients succumbing to the illness and four recovering but with long-term sequelae, necessitating ongoing oxygen supplementation at home.

#### 3.2.2. The Course of COVID-19 Regarding Laboratory Results Signature

The current body of literature provides limited meaningful data on the unique characteristics of RA patients with COVID-19, particularly concerning blood test results and pulmonary imaging findings. Furthermore, our study’s patient sample displayed significant variability owing to the lack of data.

We conducted a series of tests to assess the normal distribution of the laboratory test results. Initially, we employed the Shapiro–Wilk test for numerical variables, as it is most effective for smaller sample sizes. Subsequently, non-parametric tests (Mann–Whitney U) were employed for variables with a *p*-value ≤ 0.05 in one of the groups, while parametric tests (*t*-test for independent groups) were utilized for variables where both the RA and non-RA groups yielded a *p*-value > 0.05.

According to the Mann–Whitney U test, a statistically significant difference was observed between the two groups for the following laboratory test results: lymphocytes (z = −2.71, *p* = 0.007), Mean Corpuscular Hemoglobin Concentration (MCHC) (z = −3.101, *p* = 0.02). In addition, according to the t-test for independent groups, a statistically significant difference was observed between the two groups in terms of the average values of ESR (t = 2.85, *p* = 0.007) and Red Cell Distribution Width (RDW) (t = 2.120, *p* = 0.040), with higher values noted in the RA group for both laboratory test results. The detailed data are available for reference in Appendix A, Appendix B and Appendix C and the summarized information for the Mann–Whitney U test is presented in Table 3.

Our assessment of pulmonary radiographs and computed tomography (CT) scans revealed seventeen cases of interstitial pneumonia and four instances of alveolar pneumonia in the RA group. Additionally, four cases displayed ground-glass opacities, and one case showed pulmonary embolism. In contrast, the control group exhibited five cases of interstitial pneumonia, eight cases of alveolar pneumonia, one case of pleurisy, and 15 instances of ground-glass opacities.

During the COVID-19 pandemic, the healthcare system was impacted by the reduced availability and accessibility of various medical services, including imagistic investigations. The heightened demand for critical care, combined with the necessity to minimize exposure risks, led to a considerable reduction in the capacity to perform routine and even necessary imaging procedures. Consequently, the data available regarding pulmonary imaging of COVID-19 patients during this period is limited. Additionally, the imaging performed may have been biased toward more severe cases, given the prioritization of resources for critically ill patients. Therefore, it is essential to consider these limitations when assessing the pulmonary imaging findings of COVID-19 patients from this period.

#### 3.2.3. The Course of COVID-19 Regarding Treatment Regimens

We observed a comprehensive array of SARS-CoV-2 treatments in our groups tailored to address the varied manifestations and severities of the infection.

The therapeutic options included antivirals, mainly nucleoside and nucleotide analogs, and antibiotics, primarily chloroquine derivatives, to prevent or treat secondary bacterial infections. Corticosteroids, specifically Dexamethasone, were used predominantly in patients exhibiting decreased oxygen saturation (SpO_2_) to mitigate inflammation and improve respiratory function. Symptomatic treatments included a range of medications to alleviate various symptoms, such as analgesics, antitussives, antipyretics, antiemetics, and nutritional supplements like Vitamin D and C and Quercetin (also a cellular cycle modulator) to bolster the immune response. Anticoagulants, primarily low molecular weight heparin (LMWH), were administered to prevent thromboembolic events, and rehydration therapy, including saline infusions and other fluids, was employed to maintain adequate hydration.

The distribution of these treatments within the RA group was as follows: antivirals were administered to five patients, 15 patients received anticoagulants, antibiotics were provided to 16 patients, corticosteroids were administered to 18 patients, 37 patients utilized symptomatic treatments, and rehydration therapy was included in the treatment regimens as necessary. Regarding respiratory support, the severity of respiratory symptoms necessitated various levels of oxygen therapy: oxygen via nasal cannula (NC) was required by five patients, non-invasive ventilation (VNI) in the form of Continuous Positive Airway Pressure (CPAP) or Bilevel Positive Airway Pressure (BiPAP) was utilized by two patients, and orotracheal intubation (OTI) was necessary for one patient who experienced ARDS and deceased.

In contrast, among the non-RA group, antivirals were administered to 18 patients, anticoagulants to 35 patients (most of whom received this treatment due to their immobilized condition), antibiotics to 20 patients, and corticosteroids to 22 patients.

**Figure 3:**
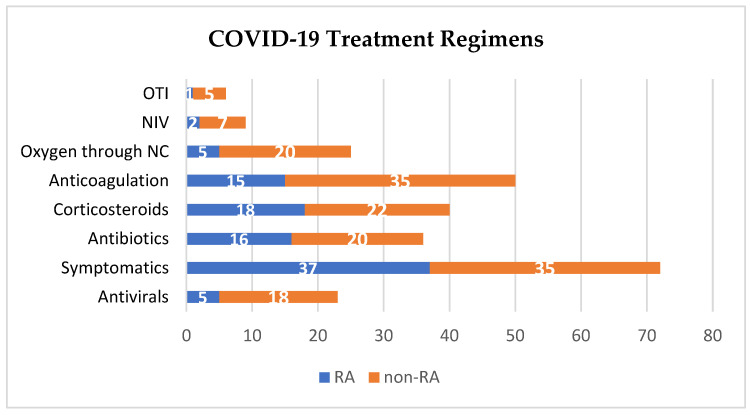
Types of COVID-19-specific treatment in the sample studied.

The results of the chi-squared test indicate a statistically significant difference between the RA and non-RA groups in terms of the COVID-19 treatments used. Detailed test values can be found in Table 4. Figure 3 illustrates the distribution of treatment within the patient sample.

These data underscore the diverse and multifaceted treatment approaches adopted by healthcare providers, reflecting the complexity of managing COVID-19 in patients with varying degrees of severity.

### 3.3. Prognosis, Outcomes, and Severity Stratification of COVID-19

Meta-analyses have shown that 17% to 31% of the general population may have asymptomatic COVID-19 infections, with no associated clinical symptoms but a positive infection test [34]. The rest of the symptomatic patients are classified as having

- Mild disease: patients with COVID-19-related symptoms without evidence of viral pneumonia or low oxygen saturation (SpO_2_);
- Moderate disease: non-severe pneumonia and SpO_2_ > 90% in room air;
- Severe disease: severe pneumonia and SpO_2_ < 90% in room air;
- Critical disease: ARDS, sepsis, septic shock, acute thrombosis [34].

Our findings indicate that the COVID-19 cases in the studied groups exhibited varying degrees of severity, with 42 instances of mild infection (26 in RA group, 16 in non-RA group), 11 cases of moderate illness (six in RA group, five in the control group), 16 cases of severe sickness (six in RA group, 10 in non-RA group), and seven cases classified as critical forms of COVID-19 (two in RA group, five in non-RA group).

Among the entire sample group, 23 individuals with RA and 14 without RA required hospitalization. Additionally, 17 non-RA cases were already hospitalized at the time of COVID-19 diagnosis, thus being unclear whether hospitalization was specifically linked to the infection.

#### 3.3.1. Is There a Definite Risk Factor for Worse COVID-19 Regarding Comorbidities?

Our study sample comprised cases with a broad range of comorbidities. Specifically, it comprised 59 individuals with cardiovascular comorbidities (HTN, ischemic heart disease, arrhythmias, heart block, valvular diseases, cardiac failure, peripheral artery disease), distributed between 25 patients in the RA group and 34 in the non-RA group. There were 43 cases with metabolic and endocrine disorders (DM, obesity, dyslipidemia, thyroid pathology), including 16 in the RA group and 27 in the control group. Pulmonary comorbidities like fibrosis, ILD, asthma, or chronic obstructive pulmonary disease (COPD) were observed in 23 cases, with 14 in the RA group and nine in the non-RA group. Reno-urinary comorbidities (chronic kidney disease, benign prostatic hyperplasia, or renal microlithiasis) were present in 14 patients, with eight in the RA group. This also included 15 cases with psychiatric comorbidities (anxiety, depression), of which three were in the RA group. Musculoskeletal comorbidities, excluding RA (and any other IMID), were noted in 53 individuals, with 25 belonging to the study group. Finally, neurological comorbidities (stroke, vertebral, or medullar trauma, epilepsy, Parkinson’s Disease, etc.) were identified in 15 patients, all of whom were in the non-RA group.

When analyzing the two groups using the chi-squared test for categorical variables, we observed that there was no significant statistical difference between the major categories of comorbidities, except neurological comorbidities in both groups (χ^2^ = 18.169, *p* = 0.000). Furthermore, when analyzing data for specific diseases, significant statistical disparity was noted for thyroid pathology (χ^2^ = 3.888, *p* = 0.049), asthma (χ^2^ = 5.309, *p* = 0.021), osteoporosis (χ^2^ = 7.679, *p* = 0.006)—all three being more frequent in the RA group—and heart blocks (χ^2^ = 6.081, *p* = 0.014), chronic heart failure (χ^2^ = 4.440, *p* = 0.035), depression (χ^2^ = 5.309, *p* = 0.021), fractures (χ^2^ = 11.316, *p* = 0.001), ischemic stroke (χ^2^ = 5.309, *p* = 0.021), vertebral-medullary trauma (χ^2^ = 4.195, *p* = 0.041)—more frequent in the control group.

**Figure 4:**
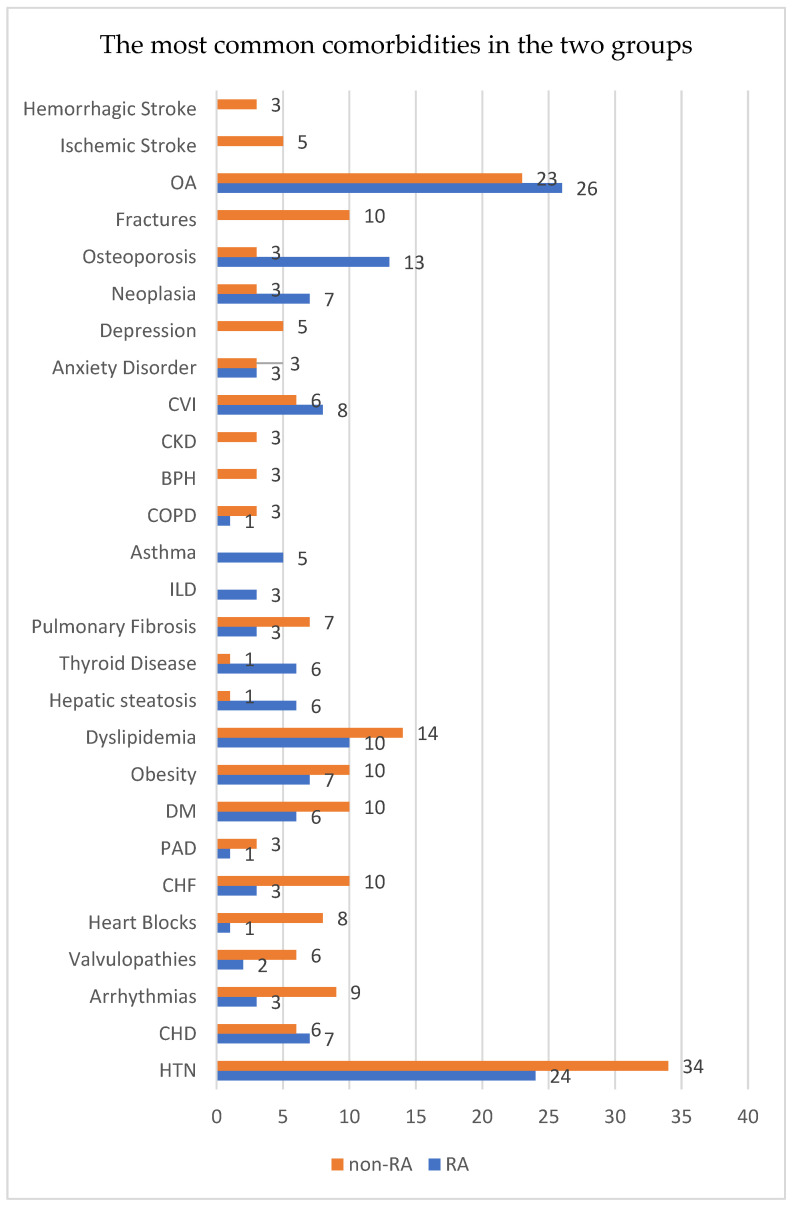
The distribution of the most common comorbidities in our groups. BPH: benign prostate hyperplasia; CHD: coronary heart disease; CHF: chronic heart failure; COPD: chronic obstructive pulmonary disease; CKD: chronic kidney disease; CVI: chronic venous insufficiency; DM: diabetes mellitus; ILD: interstitial lung disease; PAD: peripheral artery disease; OA: osteoarthritis.

The detailed and most common comorbidities are depicted in Figure 4.

In our RA study sample, there were eight cases classified as severe and critical, all of which presented with various comorbidities. Among these cases, seven were associated with CVD. Specifically, seven cases exhibited HTN, two had ischemic heart disease, and one had atrial fibrillation. Additionally, two cases had DM, one of which necessitated insulin treatment and only one patient was obese. Furthermore, three cases were linked to pulmonary comorbidities—one had a history of tuberculosis and asthma, one had pulmonary fibrosis, and another had ILD. Three patients also had osteoarthritis, and two of them had neoplasia. Notably, no psychiatric diseases or osteoporosis occurrences were detected.

Interestingly, the RA patient who passed away from the infection did not have CVD or DM. However, the patient did suffer from pulmonary fibrosis, cervical spondylosis, and recurrent urinary lithiasis. It is important to note that the aforementioned patient presented to medical professionals 20 days following the initial diagnosis of COVID-19, at which point their SpO_2_ level was recorded at 75%. Despite the late presentation, the patient was promptly admitted to the ICU and remained in care for another month.

In the control group, a total of 15 severe and critical COVID-19 cases were identified, all of which presented with a range of comorbidities. The most frequent comorbidities were cardiovascular diseases, observed in 14 of these patients. Specifically, hypertension was present in all 14 cases, ischemic heart disease in two cases, arrhythmias in four cases, and heart blocks in nine cases. Musculoskeletal disorders were the second most common comorbidity, with osteoarthritis documented in 10 patients. Metabolic and endocrine disorders were also significant, affecting nine patients. Among these, three patients had diabetes mellitus that did not require insulin therapy, three were classified as obese, and six exhibited dyslipidemia. Pulmonary conditions were identified in seven patients, with pulmonary fibrosis noted in five cases and chronic obstructive pulmonary disease (COPD) in three cases. Additionally, two patients had a history of neoplastic disease, and another two had experienced a recent ischemic stroke. Tragically, three patients in this group succumbed to COVID-19. All of these patients had multiple comorbidities, including various cardiovascular conditions, pulmonary fibrosis, COPD, and osteoarthritis.

The study aimed to determine whether there was a statistically significant difference in the frequency of comorbidities and the severity of COVID-19 among patients. The analysis yielded a lack of statistical significance in this matter.

#### 3.3.2. RA Flares and COVID-19

We also analyzed the course of SARS-CoV-2 evolution regarding RA activity during and after infection (within the first 3 months). Thus, in our study group, during COVID-19, according to the average DAS28CRP, it was found that RA was in remission in five cases, had low activity in 19 cases, moderate activity in 13 cases, and high activity in two instances. From the analysis of RA status up to 3 months post-infection, it was observed that a vast number—32 out of the total 43 cases—experienced an RA flare-up, which necessitated a change in baseline treatment in 19 cases, both csDMARDs and bDMARDs or required pulse therapy with glucocorticoids. These data highlight the intense immune activation triggered by SARS-CoV-2, caused by the engagement of proinflammatory cytokines (primarily, but not limited to, TNF-α, IL-1, IL-6) and the activation of the immune cascade [2,11].

#### 3.3.3. Different RA Treatment Regimens and Their Impact

**Figure 5:**
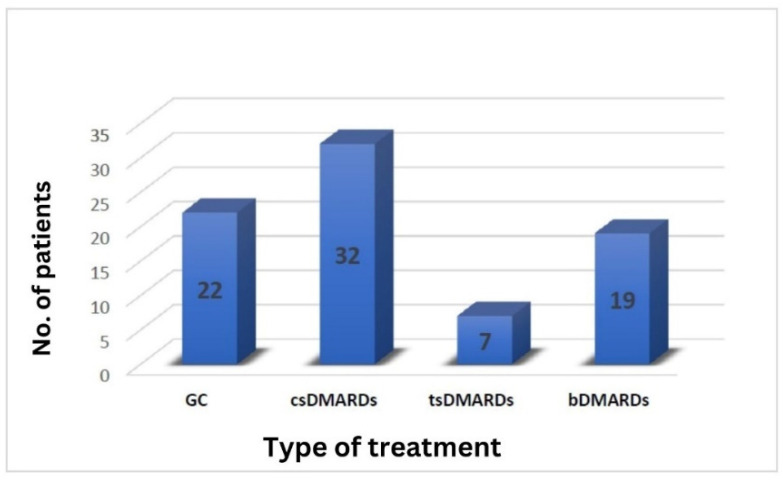
Distribution of RA treatment in our study group.

Figure 5 depicts the available medication for the patients in our study group. Corticosteroid treatment was utilized in 22 instances, but only in one case with a daily PDN dosage of 20 mg. In contrast, in the remaining 21 cases, patients were reliant on corticosteroids, with daily PDN doses ≤10 mg, underscoring an unmet need in patients with IMIDs in general, regardless of the infection status.

In 32 cases, csDMARDs were utilized. Hydroxychloroquine (HCQ) was administered in four cases at a daily dosage of 400 mg. It is important to note that despite the prior belief in the effectiveness of HCQ treatment for COVID-19, no patient with RA was prescribed this therapy upon contracting the infection. Nine patients received Leflunomide at a daily dose of 20 mg, while the remaining patients received varying weekly doses of Methotrexate (MTX) tailored to individual tolerance and response.

Seven cases involved the use of tsDMARDs, with Tofacitinib, Baricitinib, and Upadacitinib utilized in roughly equal proportions.

Biological DMARDs were prescribed in 19 cases. Among these, 13 patients were administered TNF-α inhibitors, five received RTX, and one was treated with Tocilizumab (TCZ). Noteworthy is that nine of these patients were undergoing their first biologic treatment, while four cases were classified as “difficult to treat,” necessitating at least their third biologic treatment.

#### 3.3.4. Our Research Group and Severity of COVID-19

Furthermore, in addition to the previously mentioned comorbidities and the duration of RA, we conducted various other tests to establish the statistical significance between different severity grades.

In the RA group, there were six cases of severe COVID-19 and two critical cases, including one fatality. The female-to-male ratio for these eight cases was 6:2, with an average age of 64.5 years (exceeding the average age of the entire RA group), and the average duration from RA diagnosis to infection was 11.75 years, compared to the group average of 10.53 years.

Pertaining to the patient’s smoking status, five individuals reported never having smoked, while one female patient had discontinued smoking one year prior.

With respect to the baseline treatment, four patients were corticosteroid-dependent, four were undergoing treatment with MTX, and three with RTX, with varying durations from the last infusion (14 days to 1 year). All eight patients exhibited moderately increased RA activity, as evidenced by the DAS28CRP score at the time of infection. The majority had cardiovascular comorbidities.

Conversely, in the non-RA group, there were nine severe and six critical COVID-19 cases, with a female-to-male ratio of 11:4 and an average age of 77. All of these cases had respiratory symptomatology. The predominant comorbidities were cardiovascular (14 cases), musculoskeletal (other than an IMID) in 10 cases, metabolic (nine cases), and pulmonary (seven cases). A summary of these characteristics is presented in Table 5.

To accurately characterize and establish correlations between the severity of COVID-19 within both groups (study and control), our analysis was focused exclusively on the severe and critical forms of SARS-CoV-2. For this purpose, based on the related specific statistical analysis described in the materials and method section, we observed significant statistical differences (ANOVA test) between severe and critical forms of COVID-19 in the two groups. Specifically, there was a positive correlation with age, ESR, leucocytes, neutrophils, NLR, AST, serum creatinine, serum urea, and neurological comorbidities. Conversely, we observed a negative correlation with SpO_2_, lymphocytes, and MCHC.

We focused our research further on the characterization of the RA group. The analysis of SARS-CoV-2 infection severity based on patient sex indicates that female patients experienced 22 cases of mild infections, seven cases of moderate infections, three cases of severe infections, and one critical case. In contrast, male patients encountered five mild cases, one severe, and one critical case. It is important to note that the observed variances did not yield statistically significant differences (χ^2^(4) = 3.936, *p* = 0.415).

ANOVA test was performed to assess the potential variations in the duration of COVID-19 symptoms based on the severity of infection (Table 6). The results showed a progressive increase in symptom duration from mild disease (8.36 ± 6.06 days) to moderate disease (33.71 ± 64.63 days) and to severe disease (48.50 ± 36.27 days). These differences in symptom duration among the severity groups were statistically significant (F = 8.128, *p* < 0.001). Furthermore, our results demonstrated that in the context of COVID-19 severity, there is a statistically significant difference in serum urea levels (F = 7.528, *p* < 0.018). Elevated urea levels indicate increased patient dehydration and are incorporated in the severity scoring of pneumonia, notably the CURB-65 score, which considers serum urea levels >19 mg/dl. Furthermore, elevated serum urea is identified as a risk factor for mortality in COVID-19 patients, independent of the presence of RA [35,36].

In our analysis, we examined the correlation between the progression of RA and the severity of SARS-CoV-2 infection. Among the six cases where RA flare-ups coincided with COVID-19, four were observed in individuals with mild infection, one in a patient with moderate SARS-CoV-2, and one with severe COVID-19. It is worth noting that these variations did not exhibit statistical significance (χ^2^(4) = 1.927, *p* = 0.749).

## 4. Discussion

The results of this study underscore the impact of COVID-19 on patients with RA, elucidating the interplay between rheumatological disease and SARS-CoV-2 infection.

The average age of the patients in our study was 57.81 ± 9.07 years, the typical onset age of RA. In contrast, the median age of the control group was higher: 67.65 ± 14.65 years. Statistically significant differences between the two groups were observed for gender (χ2:5.780, *p* = 0.016) and age (z = −4.130, *p* < 0.001). The prevalence and demographic characteristics observed in our study group align with global data on RA, which predominantly affects females [17,37,38]. Additionally, the control group consisted of patients who had experienced strokes and/or spinal cord injuries following various polytraumas or, respectively, vertebral disc herniations, potentially accounting for the observed male gender predominance and older age.

The primary outcome of our study was to assess the impact of COVID-19 on the clinical and paraclinical progression in patients with RA, based on a customized data set, comparatively with patients without RA or any disease that could influence their inflammatory and immunological status.

The symptomatology of COVID-19 in both groups mirrored general COVID-19 symptoms, with respiratory, systemic, musculoskeletal, and neurological manifestations being predominant. Interestingly, the control group exhibited a higher prevalence of respiratory and systemic manifestations than the RA group. This spectrum of clinical manifestations is consistent with other studies involving RA patients [39,40]. Conversely, the study group displayed a greater incidence of neurological, musculoskeletal, and gastrointestinal symptoms than the control group. Notably, these variances demonstrated statistical significance when analyzed using the chi-squared test. Given the distinct pathophysiological backgrounds of the two groups, the observed differences in symptomatology are largely consistent with expectations. Thus, we anticipated RA patients to show more musculoskeletal symptoms, as pain in RA patients is multifactorial [10]. Rather surprisingly, the greater frequency of neurological symptoms in this group could be attributed to the permeation of the pro-inflammatory cytokines across the blood–brain barrier. The elevated levels of these molecules, particularly in the context of COVID-19, may contribute to cognitive dysfunction. For the control group, we anticipated a higher occurrence of breathing and systemic symptoms. This is understandable given that neurological patients, especially those who have experienced strokes and/or spinal cord injuries, often have compromised respiratory function.

The occurrence of prolonged symptoms in a subset of our patients is particularly noteworthy. We reported persistent symptoms such as fatigue, dyspnea, cough, and musculoskeletal pain, consistent with the concept of “long COVID” described in broader populations. These prolonged symptoms can even be persistent for an indefinite period of time (1.5–2% of patients) and significantly impact the quality of life, requiring ongoing medical attention [41,42,43].

In relation to the paraclinical test results, we carried out the Shapiro–Wilk test to evaluate the data distribution. For non-normally distributed data, the Mann–Whitney U test revealed statistically significant differences between the two groups in several key laboratory parameters. Specifically, lymphocyte counts were significantly higher in the RA group (z = −2.71, *p* = 0.007), which is consistent with the exacerbated immune response typically seen in chronic inflammatory conditions like RA. In contrast, MCHC values were significantly lower in the study group (z = −3.101, *p* = 0.02), hypochromia being frequently present in patients with underlying chronic inflammation and the presence of iron deficiency (which was not evaluated in our study) [44,45,46,47]. Moreover, while lacking statistical significance, it is noteworthy that the MCV and MCH values appeared lower in the RA group (for further details, refer to Appendix B and Appendix C), and the hemoglobin levels were observed to be at the lower threshold, indicating the influence of chronic inflammation on hematological profiles.

Additionally, for variables that followed a normal distribution, the independent t-test demonstrated significant differences in the mean values of ESR (t = 2.85, *p* = 0.007) and RDW (t = 2.120, *p* = 0.040), with both markers being elevated in the RA group. These findings are not unexpected, as both ESR and RDW are well-established inflammatory markers that tend to be elevated in chronic inflammatory diseases like RA, reflecting ongoing systemic inflammation and variability in red blood cell morphology, respectively [44,48,49].

In our examination of imagistic studies, it is important to acknowledge the overall scarcity of data as not all patients underwent imagistic investigation. These investigations may have been prioritized for more severe cases due to limited resources, therefore requiring careful interpretation of the actual data. Nevertheless, our findings revealed frequent interstitial pneumonia on pulmonary radiographs and ground glass opacities on pulmonary CT, consistent with the data found in the literature [50,51].

In the examination of COVID-19 treatment utilized in the two groups, it was observed that a higher number of patients in the control group required various forms of oxygen supplementation, anticoagulation (which may have been a prerequisite due to their immobilized state), as well as antivirals and antibiotics. These discrepancies in SARS-CoV-2 treatment were statistically significant and can be explained by the availability of individualized treatment protocols for each patient, which were implemented at the two hospitals from where the patients were selected.

We further focused our group characterization on comorbidities, knowing from the literature that some specific diseases are linked to severe COVID-19 [1,14,52]. Even though the array of comorbidities was very diverse, there was no significant statistical difference between the major categories of comorbidities between the two groups, except for neurological comorbidities (χ^2^ = 18.169, *p* = 0.000)—more frequent in the non-RA group. Nonetheless, when analyzing specific diseases, a significant statistical disparity was noted for heart blocks, cardiac failure, thyroid pathology, asthma, renal microlithiasis, depression, osteoporosis, fractures, ischemic stroke, and vertebral-medullary trauma. More specifically, as anticipated and in accordance with related literature data, thyroid pathology, asthma, and osteoporosis were more frequent in the RA group [53,54,55,56].

The secondary outcomes aimed to identify predictive risk factors for severe COVID-19 in RA patients and assess RA immunosuppressive therapies’ interference and the infection’s severity. Our study revealed no statistically significant difference in comorbidities between the two groups and between severe and critical cases, except neurological comorbidities, observed exclusively in the control group (F = 11.584, *p* = 0.000). It is essential to acknowledge that this finding may be influenced by the inclusion of neurological patients in the non-RA group, who were admitted to the Neurorehabilitation Clinic following various physical traumas or strokes. However, these patients accounted for only 15 of the 43 infection cases. Another important note is that CVD was more frequent in severe and critical forms of COVID-19 (in seven of eight RA cases and 14 of 15 non-RA cases), although no statistical significance was noted.

Furthermore, there was a positive correlation between severe COVID-19 and age and specific laboratory parameters (ESR, leukocytes, neutrophils, NLR, AST, serum creatinine, and urea). Conversely, we observed a negative correlation with SpO_2_, lymphocytes, and MCHC. The positive correlation between age and severe SARS-CoV-2 outcomes is well-documented. Additionally, the paraclinical findings associated with severe COVID-19 illustrate a hyper-inflammatory response, immune dysregulation, and respiratory distress in these patients. Additional focus was put on severe and critical cases of COVID-19 only in RA patients. Thus, we observed eight such cases with one fatality. The detailed clinical progression included various symptoms, persistent complications, and varying degrees of disease activity measured by DAS28-CRP scores. Laboratory tests highlighted an elevated serum urea level as a significant factor associated with severe COVID-19.

Following the infection, another promising finding emerged concerning the primary outcome. It was noted that a significant proportion of patients (32 out of 43) experienced flares of RA, leading to modifications in baseline treatments in 19 cases. These data indicate a significant impact of COVID-19 on the disease activity of RA patients, underscoring the potential for SARS-CoV-2 to exacerbate underlying autoimmune conditions.

Additionally, the study found that different RA treatments had varied impacts on the severity of COVID-19. Notably, corticosteroid dependence and treatment with RTX were common among severe cases, although with no statistical significance. In accordance with the literature data, RTX can weaken the defense capacity of RA patients [2,52,57,58,59]. Of the five patients who were treated with this therapy, two suffered severe forms, and one patient, a critical form, which culminated in his death. Additionally, in the group with persistent COVID-19 symptoms, three out of five patients were treated with RTX. Nonetheless, it is essential to highlight that RTX is more commonly used as a second-line therapy in patients unresponsive to other immunosuppressive therapies with long disease duration and who are likely to have associated comorbidities. In exceptional cases, RTX can be used as a first-line therapy. In this context, it is pertinent to emphasize that the more severe progression observed in patients undergoing this therapy may also be attributed to potential comorbidities, the prolonged duration of the disease, and the refractory nature of RA, which favors immune inflammation.

### 4.1. Limitations

The study’s small sample size limits the generalizability of the findings and the ability to conduct more robust statistical analyses. Additionally, as a retrospective observational study, it is subject to inherent biases such as selection and information bias. The study is further limited by the lack of adjustments for potential confounding variables. As a result, the observed differences may reflect not only the presence of RA but also the influence of other unaccounted factors. Furthermore, conducting the study in two hospitals limits the results’ applicability to other settings or populations.

### 4.2. Additional Considerations

Despite limitations, the study’s approach to data collection was thorough, utilizing medical records and direct patient interactions. The study captured a wide array of symptoms, laboratory test results, comorbidities, and treatment regimens, providing a holistic view of the impact of COVID-19 on RA patients.

This study marks an initial step in characterizing RA patients within a larger cohort of patients with IMIDs among Romanian patients as part of my PhD research. Future efforts will focus on expanding the sample size. The Ethics Committee of Colentina Clinical Hospital (No. 19/18.07.2024) has granted ethical approvals for this expansion. Further attempts to better characterize this specific group of patients may yield valuable insights into the existing literature.

## 5. Conclusions

This study provides valuable insights into the clinical, laboratory, and epidemiological characteristics of COVID-19-infected patients in two independent groups from Romania: with a diagnosis of RA and without any autoimmune condition. The data reveal that RA patients can be at a heightened risk of severe COVID-19 outcomes, prolonged symptoms, and disease flare-ups post-infection.

A notable observation is the distinct symptomatology between the two groups, with RA patients more frequently exhibiting neurological, musculoskeletal, and gastrointestinal symptoms. Moreover, prolonged symptoms were significant in both groups, impacting the quality of life and necessitating ongoing medical attention.

Additionally, the study identified significant laboratory differences, underscoring the chronic inflammatory state inherent in RA and exacerbated during COVID-19.

Furthermore, the study underscored the potential impact of RA treatments, particularly RTX, on the severity of COVID-19. Although RTX was associated (but not with statistical significance) with more severe COVID-19 outcomes, this may be attributed to the more advanced RA stage and comorbidities in patients requiring such therapy. Diseases such as cardiovascular and pulmonary conditions necessitate a multidisciplinary approach to care.

Overall, the data from our study contribute to the growing body of knowledge on the intersection of autoimmune and infectious diseases, providing a foundation for improving patient care and outcomes in this vulnerable population during and beyond the COVID-19 pandemic. Future research could fruitfully explore this issue, focusing on more extensive, multicentric studies to validate these findings and provide more robust data on the optimal management strategies for RA patients with COVID-19, endeavoring to be fulfilled in a related additional, further work.

## Figures and Tables

**Table 1:** Demographics and phenotype of the study and control groups.

Demographics and Phenotype	RA Group (n = 43)	Non-RA Group (n = 43)
Female, n	36	26
Age at the time of infection in years, mean ± std. dev.	57.81 ± 9.07	67.65 ± 14.65
Urban, n	34	35
Smoking status, n		
Never	28	35
Past	8	8
Current	7	7
SARS-CoV-2 method of diagnosis		
RT-PCR	20	37
Rapid Antigen Test	23	6
RF: tested and positive, n	35	N/A
ACPA: tested and positive, n	27	N/A
Steinbrocker’s classification		
Stage I	1	N/A
Stage II	24	N/A
Stage III	10	N/A
Stage IV	2	N/A
Disease duration in years	10.53	N/A

N/A: not applicable.

**Table 2:** The chi-squared test for COVID-19 clinical manifestations.

Variable	RA (n = 43)		Non-RA (n = 43)		χ^2^	df	*p*
	n	%	n	%			
Musculoskeletal symptoms	16	37.20%	2	4.65%	78.889	2	<0.0001
Respiratory symptoms	27	62.79%	36	83.72%	24.286	2	<0.0001
Gastrointestinal symptoms	5	11.62%	2	4.65%	80.286	2	<0.0001
Neurological symptoms	13	0.3%	7	16.27%	67.800	2	<0.0001
Systemic symptoms	20	46.51%	26	60.46%	40.783	2	<0.0001

**Table 3:** The characterization of biological markers in our research group.

Variable	Group	Median	U	Z	*p*
CRP (mg/L)	RA	27.27	48.000	−0.075	0.941
	non-RA	47.8			
Leukocytes (×10^3^/μL)	RA	8.82	199.500	−0.733	0.464
	non-RA	7.9			
*Lymphocytes* (×10^3^/μL)	*RA*	*1.65*	*114.500*	*−2.71*	*0.007*
	*non-RA*	*1.1*			
Neutrophils (×10^3^/μL)	RA	5.72	209.000	−0.512	0.609
	non-RA	5.27			
Eosinophils (×10^3^/μL)	RA	0.05	191.500	−0.931	0.352
	non-RA	0.07			
Erythrocytes (×10^3^/μL)	RA	4.39	165.500	−1.524	0.128
	non-RA	4.18			
Mean Corpuscular Volume (MCV) (fl)	RA	87.6	181.500	−1.152	0.249
	non-RA	90.9			
Mean Corpuscular Hemoglobin (MCH) (pg)	RA	28.6	149.500	−1.896	0.058
	non-RA	30.4			
*MCHC* (g/dL)	*RA*	*32.7*	*98.000*	*−3.101*	*0.02*
	*non-RA*	*33.5*			
NLR	RA	2.69	206.500	−0.570	0.569
	non-RA	4.43			
SII	RA	558.36	151.000	−0.827	0.408
	non-RA	1176			
AST (U/L)	RA	25	156.500	−1.290	0.197
	non-RA	34			
Alanine Aminotransferase (ALT) (U/L)	RA	28	170.000	−1.290	0.197
	non-RA	34			
Creatine Kinase (CK) (U/L)	RA	30	41.000	−0.855	0.392
	non-RA	58			
CK—MB (U/L)	RA	12.9	17.000	−0.916	0.360
	non-RA	15			
Serum Creatinine (mg/dL)	RA	0.84	176.000	−1.281	0.200
	non-RA	0.8			
Serum Urea (mg/dL)	RA	35.16	126.500	−0.866	0.386
	non-RA	34			
Prothrombin time (PT) (s)	RA	13.05	50.500	−1.678	0.093
	non-RA	14.85			
International Normalized Ratio (INR)	RA	1.27	79.500	−0.447	0.655
	non-RA	1.2			

Statistically significant differences observed between the two groups, with a probability value of *p* < 0.05, are represented in italics.

**Table 4:** The chi-squared test regarding COVID-19 treatment regimens in our groups.

Variable	RA (n = 43)		Non-RA (n = 43)		χ^2^	df	*p*
	n	%	n	%			
Antivirals	5	11.62%	18	41.86%	70.348	2	<0.0001
Symptomatic treatment	37	86.04%	35	81.39%	9.600	2	0.008
Antibiotics	16	37.20%	20	46.51%	50.444	2	<0.0001
Corticosteroids	18	41.86%	22	51.16%	46.400	2	<0.0001
Anticoagulants	15	34.88%	35	81.39%	44.000	2	<0.0001
Oxygen through NC	5	11.62%	20	46.51%	70.000	2	<0.0001
NIV	2	4.65%	7	16.27%	79.778	2	<0.0001
OTI	1	2.32%	5	11.62%	82.667	2	<0.0001

**Table 5:** Characteristics of severe and critical forms of COVID-19 in the study group.

Characteristics of Severe and Critical Forms of COVID-19	RA (n = 8)	Non-RA (n = 15)
Female, n	6	11
Age at the time of infection in years, mean	64.5	77
Smoking status, n		
Never	7	15
Past	1	0
COVID-19 symptomatology:		
Respiratory	6	15
Musculoskeletal	2	2
Neurological	3	4
RA duration in years, mean	11.75	N/A
Comorbidities		
Cardiovascular	7	14
Pulmonary	4	7
Metabolic	4	9
Neurological	0	3
Psychiatric	0	3
Musculoskeletal (other than an IMID)	3	10
Neoplasia	2	2
Glucocorticoids	4	N/A
MTX	4	N/A
RTX	3	N/A
RA flare in the first 3 months after COVID-19	5	N/A

N/A: not applicable.

**Table 6:** Results of the ANOVA test regarding the severity of COVID-19.

Variable	Mild COVID-19 (Median ± S.D.)	Moderate COVID-19 (Median ± S.D.)	Severe + Critical COVID-19 (Median ± S.D.)	F	*p*
Age	56.47 ± 9.31	58.14 ± 9.35	64.17 ± 4.95	2.397	0.068
Disease duration (days)	8.36 ± 6.06	33.71 ± 64.63	48.50 ± 36.27	8.128	<0.001
Serum Urea (mg/dL)	15.49 ± 3.62	36.60 ± 9.83	46.00 ± 0.00	7.528	0.018

## Data Availability

The data presented in this study are available on request from the corresponding author due to legal and ethical reasons.

## Supplementary Materials

The following supporting information can be downloaded at: https://www.mdpi.com/article/10.3390/biomedicines12092145/s1, Table S1. STROBE Statement.

## Abbreviations

ACPA	Anti-citrullinated protein antibodies
ANOVA	Analysis of variance
BiPAP	Bilevel Positive Airway Pressure
CI	Confidence interval
COPD	Chronic obstructive pulmonary disease
CRP	C-reactive protein
csDMARD	Conventional synthetic disease-modifying antirheumatic drug
DMARD	Disease-modifying antirheumatic drug
HTN	Hypertension
ICU	Intensive care unit
IMID	Immune-mediated inflammatory disease
MCH	Mean Corpuscular Hemoglobin
MTX	Methotrexate
NSAID	Nonsteroidal anti-inflammatory drug
PDN	Prednisone
RA	Rheumatoid arthritis
RT-PCR	Reverse transcription polymerase chain reaction
SD	Standard deviation
TCZ	Tocilizumab
VNI	Non-invasive Ventilation
AST	Aspartate Aminotransferase
aOR	Adjusted odds ratio
bDMARD	Biologic disease-modifying antirheumatic drug
CK	Serum Creatine Kinase
COVID-19	Coronavirus Disease 2019
CT	Computed tomography
DAS28CRP	Disease Activity Score-28 with CRP
GC	Glucocorticoid
IBD	Inflammatory Bowel Disease
IL	Interleukin
INR	International Normalized Ratio
MCHC	Mean Corpuscular Hemoglobin Concentration
NC	Nasal Cannula
OR	Odds ratio
PLT	Platelets
RDW	Red Cell Distribution Width
RTX	Rituximab
SpO_2_	Peripheral capillary oxygen saturation
TNF-α	Tumor necrosis factor-alpha
χ2	Chi-squared
ALT	Alanine Aminotransferase
ARDS	Acute respiratory distress syndrome
CD-20	Cluster of Differentiation 20
CK-MB	Serum Creatine Kinase—MB fraction
CPAP	Continuous Positive Airway Pressure
CVD	Cardiovascular disease
DM	Diabetes mellitus
HCQ	Hydroxychloroquine
IBM	International Business Machines
ILD	Interstitial lung disease
LMWH	Low molecular weight heparin
MCV	Mean Corpuscular Volume
NLR	Neutrophil-to-lymphocyte ratio
OTI	Orotracheal Intubation
PT	Prothrombin time
RF	Rheumatoid factor
SARS-CoV-2	Severe acute respiratory syndrome coronavirus 2
SPSS	Statistical Package for the Social Sciences
tsDMARD	Targeted synthetic disease-modifying antirheumatic drug
WHO	World Health Organization

## Appendix A. Test of Normality Shapiro–Wilk for Assessing the Normal Distribution of the Laboratory Test Results

**Variable (Values of Laboratory Results)**	**Group**	**W**	**df**	**Sig.**
CRP (mg/L)	RA	0.804	14	0.006
	non-RA	0.792	7	0.034
ESR (mm/h)	RA	0.924	9	0.426
	non-RA	0.945	32	0.101
Fibrinogen (mg/dL)	RA	0.816	4	0.134
	non-RA	0.955	28	0.266
Serum Hemoglobin (g/dL)	RA	0.919	14	0.215
	non-RA	0.972	33	0.539
Leukocytes (×10^3^/μL)	RA	0.866	14	0.037
	non-RA	0.943	33	0.082
Lymphocytes (×10^3^/μL)	RA	0.910	14	0.157
	non-RA	0.892	33	0.003
Neutrophils (×10^3^/μL)	RA	0.855	14	0.026
	non-RA	0.899	33	0.005
Monocytes (×10^3^/μL)	RA	0.946	14	0.498
	non-RA	0.938	33	0.061
Eosinophils (×10^3^/μL)	RA	0.855	14	0.026
	non-RA	0.846	33	<0.001
Erythrocytes (×10^3^/μL)	RA	0.980	14	0.973
	non-RA	0.177	33	<0.001
MCV (fl)	RA	0.965	14	0.809
	non-RA	0.919	33	0.017
MCH (pg)	RA	0.929	14	0.291
	non-RA	0.889	33	0.003
MCHC (g/dL)	RA	0.898	14	0.103
	non-RA	0.900	33	0.005
Hematocrit	RA	0.898	14	0.107
	non-RA	0.975	33	0.615
RDW %	RA	0.907	14	0.142
	non-RA	0.951	33	0.144
Platelets (PLT) (×10^3^/μL)	RA	0.938	11	0.501
	non-RA	0.934	33	0.046
NLR	RA	0.321	14	<0.001
	non-RA	0.731	33	<0.001
SII	RA	0.391	11	<0.001
	non-RA	0.396	33	<0.001
AST (U/L)	RA	0.567	13	<0.001
	non-RA	0.773	32	<0.001
ALT (U/L)	RA	0.760	14	0.002
	non-RA	0.875	32	0.001
CK (U/L)	RA	0.694	4	0.010
	non-RA	0.395	28	<0.001
CKMB (U/L)	RA	0.513	2	<0.001
	non-RA	0.513	28	<0.001
Serum Creatinine (mg/dL)	RA	0.850	14	0.022
	non-RA	0.957	33	0.213
Serum Urea (mg/dL)	RA	0.942	10	0.575
	non-RA	0.864	31	0.001
PT (s)	RA	0.695	6	0.005
	non-RA	0.758	30	<0.001
APTT (s)	RA	0.840	5	0.165
	non-RA	0.954	30	0.210
INR	RA	0.657	6	0.002
	non-RA	0.763	30	<0.001

## Appendix B. Mann–Whitney U Test for Laboratory Results

**Table:**

**Variable**	**Group**	**Median**	**U**	**Z**	***p***
CRP (mg/L)	RA	27.27	48.000	−0.075	0.941
	non-RA	47.8			
Leukocytes (×10^3^/μL)	RA	8.82	199.500	−0.733	0.464
	non-RA	7.9			
*Lymphocytes* (×10^3^/μL)	*RA*	*1.65*	*114.500*	*−2.71*	*0.007*
	*non-RA*	*1.1*			
Neutrophils (×10^3^/μL)	RA	5.72	209.000	−0.512	0.609
	non-RA	5.27			
Eosinophils (×10^3^/μL)	RA	0.05	191.500	−0.931	0.352
	non-RA	0.07			
Erythrocytes (×10^3^/μL)	RA	4.39	165.500	−1.524	0.128
	non-RA	4.18			
MCV (fl)	RA	87.6	181.500	−1.152	0.249
	non-RA	90.9			
MCH (pg)	RA	28.6	149.500	−1.896	0.058
	non-RA	30.4			
*MCHC* (g/dL)	*RA*	*32.7*	*98.000*	*−3.101*	*0.02*
	*non-RA*	*33.5*			
NLR	RA	2.69	206.500	−0.570	0.569
	non-RA	4.43			
SII	RA	558.36	151.000	−0.827	0.408
	non-RA	1176			
AST (U/L)	RA	25	156.500	−1.290	0.197
	non-RA	34			
ALT (U/L)	RA	28	170.000	−1.290	0.197
	non-RA	34			
CK (U/L)	RA	30	41.000	−0.855	0.392
	non-RA	58			
CKMB (U/L)	RA	12.9	17.000	−0.916	0.360
	non-RA	15			
Serum Creatinine (mg/dL)	RA	0.84	176.000	−1.281	0.200
	non-RA	0.8			
Serum Urea (mg/dL)	RA	35.16	126.500	−0.866	0.386
	non-RA	34			
PT (s)	RA	13.05	50.500	−1.678	0.093
	non-RA	14.85			
INR	RA	1.27	79.500	−0.447	0.655
	non-RA	1.2			

Statistically significant differences observed between the two groups, with a probability value of *p* < 0.05, are represented in italics.

## Appendix C. t-Test for Laboratory Results

**Table:**

**Variable**	**Group**	**Mean** **±** **S.D.**	**U**	**Z**	***p***
*ESR* (mm/h)	*RA*	*75.11 ± 11.23*	*2.85*	*43*	*0.007*
	*Non-RA*	*43.59 ± 4.94*			
Serum fibrinogen (mg/dL)	RA	558.50 ± 221.19	−1.391	30	0.175
	Non-RA	678.32 ± 153.07			
Serum hemoglobin (g/dL)	RA	12.62 ± 1.20	0.737	45	0.233
	Non-RA	12.26 ± 1.69			
Monocytes (×10^3^/μL)	RA	0.57 ± 1.99	−0.581	45	0.564
	Non-RA	0.62 ± 0.33			
Hematocrit (%)	RA	38.77 ± 3.15	1.360	45	0.181
	Non-RA	36.74 ± 5.18			
*RDW* (%)	*RA*	*15.38 ± 2.90*	*2.120*	*45*	*0.040*
	*Non-RA*	*14.11 ± 1.24*			
PLT (×10^3^/μL)	RA	298.00 ± 132.74	0.825	42	0.414
	Non-RA	265.15 ± 108.00			
APTT (s)	RA	73.10 ± 30.62	−0.978	33	0.335
	Non-RA	85.13 ± 24.69			

Statistically significant differences observed between the two groups, with a probability value of *p* < 0.05, are represented in italics.

## References

[1] Codreanu C., Ionescu R., Predeteanu D., Rezus E., Parvu M., Mogosan C., Popescu C.C., Rednic S. (2020). Recommendations of the Romanian Society of Rheumatology regarding the management of patients with rheumatic diseases in the context of the SARS-CoV-2 pandemic. Rom. J. Rheumatol..

[2] Trandafir A.I., Onose G., Munteanu C., Băila M., Saglam A.O., Mandu M., Săulescu I., Grădinaru E., Bojincă V.-C. (2023). Particularities regarding Clinical-biological and Evolutive Parameters of Immune-mediated Rheumatic Diseases in Patients with COVID-19-systematic literature review. Balneo PRM Res. J..

[3] Robu A.M., Onose G., Ulinici M.T., Andrei R., Bălănescu A., Comănici V.D., Ciomârtan T., Codreanu I.-F. (2024). Actual data regarding the impact of viral respiratory co-infection (COVID-19 and flu/Respiratory Syncytial Virus-RSV)—A systematic review. Balneo PRM Res. J..

[4] Isnardi C.A., Roberts K., Saurit V., Petkovic I., Báez R.M., Quintana R., Tissera Y., Ornella S., DAngelo Exeni M.E., Pisoni C.N. (2023). Sociodemographic and clinical factors associated with poor COVID-19 outcomes in patients with rheumatic diseases: Data from the SAR-COVID Registry. Clin. Rheumatol..

[5] COVID—Coronavirus Statistics—Worldometer. https://www.worldometers.info/coronavirus/#countries.

[6] Brumă E., Onose G., Ciobanu V. (2023). Research on clinical-paraclinical and evolutive aspects in patients with post spinal cord injury (SCI) statuses and COVID-19—A systematic literature review. Balneo PRM Res. J..

[7] Munteanu C., Onose G., Turnea M.A., Rotariu M. (2023). Cellular and Molecular Homeostatic Microenvironmental imbalances in Osteoarthritis and Rheumatoid Arthritis. Balneo PRM Res. J..

[8] Romay-Barrero H., Herrero-López J., Llorente-González A., Corral G.M.D., Palomo-Carrión R., Martínez-Galán I. (2022). Balneotherapy and health-related quality of life in individuals with Rheumatoid arthritis: An observational study under real clinical practice conditions. Balneo PRM Res. J..

[9] England B., Mikuls T. UpToDate. 2024. Epidemiology of, Risk Factors for, and Possible Causes of Rheumatoid Arthritis. https://www.uptodate.com/contents/epidemiology-of-risk-factors-for-and-possible-causes-of-rheumatoid-arthritis.

[10] Berghea F., Berghea C.E., Zaharia D., Trandafir A.I., Nita E.C., Vlad V.M. (2021). Residual Pain in the Context of Selecting and Switching Biologic Therapy in Inflammatory Rheumatic Diseases. Front. Med..

[11] Bonek K., Roszkowski L., Massalska M., Maslinski W., Ciechomska M. (2021). Biologic Drugs for Rheumatoid Arthritis in the Context of Biosimilars, Genetics, Epigenetics and COVID-19 Treatment. Cells.

[12] Bălănescu A.R., Bălănescu Ș.M. (2019). Imunitatea Înnăscută Și Dobândită—Noțiuni Fundamentale. Autoimunitatea.

[13] Ionescu R. (2022). Esențialul în Reumatologie.

[14] Ge E., Li Y., Wu S., Candido E., Wei X. (2021). Association of pre-existing comorbidities with mortality and disease severity among 167,500 individuals with COVID-19 in Canada: A population-based cohort study. PLoS ONE.

[15] Bournia V.K., Fragoulis G.E., Mitrou P., Mathioudakis K., Tsolakidis A., Konstantonis G., Tseti I., Vourli G., Tektonidou M.G., Paraskevis D. (2023). Different COVID-19 outcomes among systemic rheumatic diseases: A nation-wide cohort study. Rheumatology.

[16] Saadoun D., Vieira M., Vautier M., Baraliakos X., Andreica I., da Silva J.A.P., Sousa M., Luis M., Khmelinskii N., Gracía J.M.A. (2021). SARS-CoV-2 outbreak in immune-mediated inflammatory diseases: The Euro-COVIMID multicentre cross-sectional study. Lancet Rheumatol..

[17] Rezus E., Popescu C.C., Mogosan C., Avram C., Ionitescu R.C., Felea I., Filipescu I.-C., Codreanu C. (2023). Treatment of rheumatoid arthritis with biologic targeted synthetic disease-modifying anti-rheumatic drugs in 2022-real-world data from the Romanian Registry of Rheumatic Diseases. Rom. J. Rheumatol..

[18] Pellegrino R., Pellino G., Selvaggi F., Federico A., Romano M., Gravina A.G. (2022). Therapeutic adherence recorded in the outpatient follow-up of inflammatory bowel diseases in a referral center: Damages of COVID-19. Dig. Liver Dis..

[19] Toori K.U., Qureshi M.A., Chaudhry A., Safdar M.F. (2021). Neutrophil to lymphocyte ratio (NLR) in COVID-19: A cheap prognostic marker in a resource constraint setting. Pak. J. Med. Sci..

[20] Li X., Liu C., Mao Z., Xiao M., Wang L., Qi S., Zhou F. (2020). Predictive values of neutrophil-to-lymphocyte ratio on disease severity and mortality in COVID-19 patients: A systematic review and meta-analysis. Crit. Care.

[21] Gelzo M., Cacciapuoti S., Pinchera B., De Rosa A., Cernera G., Scialò F., Mormile M., Fabbrocini G., Parrella R., Gentile I. (2021). Prognostic Role of Neutrophil to Lymphocyte Ratio in COVID-19 Patients: Still Valid in Patients That Had Started Therapy?. Front. Public Health.

[22] Chan A.S., Rout A. (2020). Use of Neutrophil-to-Lymphocyte and Platelet-to-Lymphocyte Ratios in COVID-19. J. Clin. Med. Res..

[23] Filip C., Covali R., Socolov D., Akad M., Carauleanu A., Andrada Vasilache I., Scripcariu I.S., Pavaleanu I., Dumachita-Sargu G., Butureanu T. (2023). The Influence of Climate on Critically Ill Pregnant COVID-19 Patients, as Revealed by the Inflammation Indexes, in Spring versus Autumn 2021 Infection. Balneo PRM Res. J..

[24] Satis S. (2021). New Inflammatory Marker Associated with Disease Activity in Rheumatoid Arthritis: The Systemic Immune-Inflammation Index. Curr. Health Sci. J..

[25] Mangoni A.A., Zinellu A. (2024). The diagnostic role of the systemic inflammation index in patients with immunological diseases: A systematic review and meta-analysis. Clin. Exp. Med..

[26] Fois A.G., Paliogiannis P., Scano V., Cau S., Babudieri S., Perra R., Ruzzittu G., Zinellu E., Pirina P., Carru C. (2020). The Systemic Inflammation Index on Admission Predicts In-Hospital Mortality in COVID-19 Patients. Molecules.

[27] Mangoni A.A., Zinellu A. (2023). Systemic inflammation index, disease severity, and mortality in patients with COVID-19: A systematic review and meta-analysis. Front. Immunol..

[28] Citu C., Gorun F., Motoc A., Sas I., Gorun O.M., Burlea B., Tuta-Sas I., Tomescu L., Neamtu R., Malita D. (2022). The Predictive Role of NLR, d-NLR, MLR, and SIRI in COVID-19 Mortality. Diagnostics.

[29] Ionescu C.E., Popescu C.C., Agache M., Dinache G., Codreanu C. (2024). Depression in Rheumatoid Arthritis: Prevalence and Effects on Disease Activity. J. Clin. Med..

[30] Kay J., Upchurch K.S. (2012). ACR/EULAR 2010 rheumatoid arthritis classification criteria. Rheumatology.

[31] IBM Corp. (2023). IBM SPSS Statistics for Windows.

[32] Váncsa S., Dembrovszky F., Farkas N., Szakó L., Teutsch B., Bunduc S., Nagy R., Párniczky A., Erőss B., Péterfi Z. (2021). Repeated SARS-CoV-2 Positivity: Analysis of 123 Cases. Viruses.

[33] Hashimoto H., Hashimoto S., Shimazaki Y. (2022). Functional Impairment and Periodontitis in Rheumatoid Arthritis. Int. Dent. J..

[34] WHO (2021). Guideline Clinical Management of COVID-19 Patients: Living Guideline, 18 November 2021. https://www.who.int/publications/i/item/WHO-2019-nCoV-clinical-2023.2.

[35] Rodrigues H.C.N., Silva M.L., Mantovani M.D.S., Silva J.M.D., Domingues M.F.P., Tanni S.É., Azevedo P.S., Minicucci M.F., Buffarah M.N.B., Pereira A.G. (2023). Higher urea-to-albumin ratio is associated with mortality risk in critically ill COVID-19 patients. Clin. Nutr. ESPEN.

[36] Yin J., Wang Y., Jiang H., Wu C., Sang Z., Sun W., Wei J., Wang W., Liu D., Huang H. (2024). Blood urea nitrogen and clinical prognosis in patients with COVID-19: A retrospective study. Medicine.

[37] Scott I.C., Whittle R., Bailey J., Twohig H., Hider S.L., Mallen C.D., Muller S., Jordan K.P. (2022). Rheumatoid arthritis, psoriatic arthritis, and axial spondyloarthritis epidemiology in England from 2004 to 2020: An observational study using primary care electronic health record data. Lancet Reg. Health Eur..

[38] George M.D., Venkatachalam S., Banerjee S., Baker J.F., Merkel P.A., Gavigan K., Curtis D., Danila M.I., Curtis J.R., Nowell W.B. (2021). Concerns, Healthcare Use, and Treatment Interruptions in Patients with Common Autoimmune Rheumatic Diseases During the COVID-19 Pandemic. J. Rheumatol..

[39] Dernoncourt A., Schmidt J., Duhaut P., Liabeuf S., Gras-Champel V., Masmoudi K., Bennis Y., Batteux B. (2021). COVID-19 in DMARD-treated patients with inflammatory rheumatic diseases: Insights from an analysis of the World Health Organization pharmacovigilance database. Fundam. Clin. Pharmacol..

[40] Malek Mahdavi A., Varshochi M., Hajialilo M., Dastgiri S., Khabbazi R., Khabbazi A. (2021). Factors associated with COVID-19 and its outcome in patients with rheumatoid arthritis. Clin. Rheumatol..

[41] Di Iorio M., Cook C.E., Vanni K.M.M., Patel N.J., D’Silva K.M., Fu X., Wang J., Prisco L.C., Kowalski E., Zaccardelli A. (2022). DMARD disruption, disease flare, and prolonged symptom duration after acute COVID-19 among participants with rheumatic disease: A prospective study. Semin. Arthritis Rheum..

[42] Diiorio M., Kennedy K., Liew J.W., Putman M.S., Sirotich E., Sattui S.E., Foster G., Harrison C., Larché M.J., Levine M. (2022). Prolonged COVID-19 symptom duration in people with systemic autoimmune rheumatic diseases: Results from the COVID-19 Global Rheumatology Alliance Vaccine Survey. RMD Open.

[43] Tăbîrță A., Bulai M., Chihai V., Pascal O. (2023). Assessment of Musculoskeletal Pain in Medical Rehabilitation of POST-COVID-19 patient. Res. J..

[44] Khorasanchi Z., Rashidmayvan M., Hasanzadeh E., Moghadam M.R.S.F., Afkhami N., Asadiyan-Sohan P., Fard M.V., Mohammadhasani K., Varaste N., Sharifan P. (2023). The association of hematological inflammatory markers and psychological function in COVID-19 patients: A cross-sectional study. Physiol. Rep..

[45] Tezcan M.E., Acer Kasman S., Şen N., Osken S., Yılmaz-Oner S. (2023). Importance of hematological markers in familial Mediterranean fever in terms of disease severity and amyloidosis. Rheumatol. Int..

[46] Choy M., Zhen Z., Dong B., Chen C., Dong Y., Liu C., Liang W., Xue R. (2023). Mean corpuscular haemoglobin concentration and outcomes in heart failure with preserved ejection fraction. ESC Heart Fail..

[47] Xia W., Jiang T., Tan Y., Li C., Wu S., Mei B. (2023). Characteristics of hematological parameters on admission in COVID-19 Omicron variant infected in Chinese population: A large-scale retrospective study. BMC Infect. Dis..

[48] Kalay N., Aytekin M., Kaya M.G., Özbek K., Karayakali M., Söǧüt E., Altunkas F., Oztürk A., Koç F. (2011). The relationship between inflammation and slow coronary flow: Increased red cell distribution width and serum uric acid levels. Turk. Kardiyol. Dern. Arsivi..

[49] Atwa E.T., Omar H.M., Amin A., Hammad M. (2022). Red cell distribution width and mean platelet volume in rheumatoid arthritis patients: Its association with disease activity. Reumatol. Clin..

[50] Balasubramaniam S., Raju B.P., Kumarasamy S.P., Ramasubramanian S., Srinivasan A.K., Gopinath I., Shanmugam K., Kumar A.S., Visakan Sivasakthi V., Srinivasan S. (2024). Lung Involvement Patterns in COVID-19: CT Scan Insights and Prognostic Implications From a Tertiary Care Center in Southern India. Cureus.

[51] Migliore M. (2021). Ground glass opacities of the lung before, during and post COVID-19 pandemic. Ann. Transl. Med..

[52] Bournia V.K., Fragoulis G.E., Mitrou P., Mathioudakis K., Konstantonis G., Tektonidou M.G., Tsolakidis A., Paraskevis D., Sfikakis P.P. (2024). Outcomes of COVID-19 Omicron variant in patients with rheumatoid arthritis: A nationwide Greek cohort study. Rheumatology.

[53] Liu Y.Q., Liu Y.Q., Chen Z.Y., Li H., Xiao T. (2021). Rheumatoid arthritis and osteoporosis: A bi-directional Mendelian randomization study. Aging.

[54] Rolfes M.C., Juhn Y.J., Wi S.I., Sheen Y.H. (2017). Asthma and the Risk of Rheumatoid Arthritis: An Insight into the Heterogeneity and Phenotypes of Asthma. Tuberc. Respir. Dis..

[55] Conigliaro P., D’Antonio A., Pinto S., Chimenti M.S., Triggianese P., Rotondi M., Perricone R. (2020). Autoimmune thyroid disorders and rheumatoid arthritis: A bidirectional interplay. Autoimmun. Rev..

[56] Figus F.A., Piga M., Azzolin I., McConnell R., Iagnocco A. (2021). Rheumatoid arthritis: Extra-articular manifestations and comorbidities. Autoimmun. Rev..

[57] Minca D., Minca A., Ionescu C.E., Dinache O.G., Popescu C., Agache M., Enache L., Mogosan C., Codreanu C. (2022). POS1205 COVID-19 in Patients with Inflammatory Rheumatic Diseases—Data from the Romanian Registry of Rheumatic Diseases. Ann. Rheum. Dis..

[58] Ekin A., Coskun B.N., Dalkilic E., Pehlivan Y. (2023). The effects of COVID-19 infection on the mortality of patients receiving rituximab therapy. Ir. J. Med. Sci..

[59] Singh N., Madhira V., Hu C., Olex A.L., Bergquist T., Fitzgerald K.C., Huling J.D., Patel R.C., Singh J.A. (2023). Rituximab is associated with worse COVID-19 outcomes in patients with rheumatoid arthritis: A retrospective nationally sampled cohort study from the, U.S. National COVID Cohort Collaborative (N3C). Semin. Arthritis Rheum..

