# Supplementary material for: Unraveling the Impact of COVID-19 on Rheumatoid Arthritis: Insights from Two Romanian Hospitals—Preliminary Results
Source: Biomedicines. 2024 Sep 21;12(9):2145. doi: 10.3390/biomedicines12092145 (PMC11430409; doi:10.3390/biomedicines12092145)
Supplement: Supplementary file 1 [file biomedicines-12-02145-s001.zip › biomedicines-3154717-supplementary.pdf]

Supplementary Table S1 - STROBE Statement—Checklist of items that should be included in reports of *case-control studies* – corresponding to the original article: "Unraveling the Impact of COVID-19 on Rheumatoid Arthritis: Insights from Two Romanian Hospitals"

	Item No	Recommendation
**Title and abstract**	1	(*a*) Indicate the study's design with a commonly used term in the title or the abstract<br>**Comment**: The title "Unraveling the Impact of COVID-19 on Rheumatoid Arthritis: Insights from Two Romanian Hospitals" appropriately identifies the study as a case-control study. *(Title, Page 1)*
		(*b*) Provide in the abstract an informative and balanced summary of what was done and what was found<br>**Comment**: The abstract currently highlights the study's aim, methods, results, discussion and conclusions. *(Abstract, Page 1, lines 24-48)*
**Introduction**		
Background/rationale	2	Explain the scientific background and rationale for the investigation being reported<br>**Comment**: The Introduction Section clearly offers a detailed perspective on COVID-19 and the rationale of investigating this infection in RA patients. *(Introduction, Pages 2-4, Lines 52-141)*
Objectives	3	State-specific objectives, including any prespecified hypotheses<br>**Comment**: The Introduction Section states the overall primary, secondary and associated outcomes. *(Introduction, Page 4, Lines 142-148)*
**Methods**		
Study design	4	Present key elements of study design early in the paper<br>**Comment**: The study design is clearly stated in the "Materials and Methods" Section as a retrospective observational case-control study. *(Materials and Methods, Page 4, Line 155)*
Setting	5	Describe the setting, locations, and relevant dates, including periods of recruitment, exposure, follow-up, and data collection<br>**Comment**: This information is detailed in the "Materials and Methods" Section. The study was conducted in two hospitals from Bucharest, Romania ("Sfânta Maria" Clinical Hospital and "Bagdasar-Arseni" Teaching Emergency Hospital), recruiting COVID-19-infected patients between March 1, 2020, and February 29, 2024. The first group consisted of patients diagnosed with rheumatoid arthritis and the second group – of patients without any immune, inflammatory or infectious disease. Information was collected from medical records/files, the hospital's database, and directly through face-to-face or telephone conversations, with prior informed consent obtained. *(Materials and Methods, Page 4, Lines 159-176)*
Participants	6	(*a*) Give the eligibility criteria, and the sources and methods of case ascertainment and control selection. Give the rationale for the choice of cases and controls<br>**Comment**: The study described the inclusion and exclusion criteria in the "Materials and Methods" Section, providing detailed descriptions of the RA and non-RA groups. *(Materials and Methods, Page 4 , Lines 159-186)*
		(*b*) For matched studies, give matching criteria and the number of controls per case<br>**Comment**: The control group matched the number of the study group and both groups consisted of COVID-19-infected patients. *(Materials and Methods, Page 4, Lines 159-176)*

Variables	7	Clearly define all outcomes, exposures, predictors, potential confounders, and effect modifiers. Give diagnostic criteria, if applicable<br>**Comment**: The study includes the main outcomes and variables, including demographic, clinical, and laboratory characteristics of the RA and non-RA groups, in the "Introduction" and "Material and Methods" Sections. The exposures are diagnostic of RA (measured and classified according to current criteria), COVID-19 (diagnosed through RT-PCR or Rapid-Antigen test), and immunosuppressive treatments like DMARDs. *(Introduction, Page 4, Lines 142-148 and Materials and Methods, Page 4, Lines 159-186)*
Data sources/ measurement	8*	For each variable of interest, give sources of data and details of methods of assessment (measurement). Describe comparability of assessment methods if there is more than one group<br>**Comment**: Data was collected from medical records and through direct patient interaction, which aligns with STROBE guidelines. *(Materials and Methods, Page 4, Lines 177-186)*
Bias	9	Describe any efforts to address potential sources of bias<br>**Comment**: As a retrospective observational study, it is subject to inherent biases such as selection bias and information bias. *(Discussions, Page 19, Lines: 706-712)*
Study size	10	Explain how the study size was arrived at<br>**Comment**: The available and complete data we had when writing the study was collected for 43 RA patients. Furthermore, we augment the data set by adding a control group of 43 non-RA patients.
Quantitative variables	11	Explain how quantitative variables were handled in the analyses. If applicable, describe which groupings were chosen and why<br>**Comment**: The study contains a detailed description of statistical analysis using SPSS and various statistical tests according to the data collected. *(Materials and Methods, Page 4-5, Lines: 187-204)*
Statistical methods	12	(*a*) Describe all statistical methods, including those used to control for confounding<br>**Comment**: The study contains a detailed description of statistical analysis using SPSS and various statistical tests according to the data collected. Due to the small sample size, no adjustments were made for potential confounding factors. *(Materials and Methods, Page 4-5, Lines: 187-214)*
		(*b*) Describe any methods used to examine subgroups and interactions<br>**Comment**: *Materials and Methods, Page 4-5, Lines: 187-214*
		(*c*) Explain how missing data were addressed<br>**Comment**: N/A
		(*d*) If applicable, explain how matching of cases and controls was addressed<br>**Comment**: Being an early-phase study, the two groups were matched only in size. This allowed us to identify potential differences that can be explored in more detail in future studies.
		(*e*) Describe any sensitivity analyses<br>**Comment**: N/A
**Results**		
Participants	13*	(a) Report numbers of individuals at each stage of study—eg numbers potentially eligible, examined for eligibility, confirmed eligible, included in the study, completing follow-up, and analysed<br>**Comment**:<br>*Materials and Methods, Page 4, Lines: 173-176; Results, Page 5, Lines: 217-226*

		(b) Give reasons for non-participation at each stage<br>**Comment**: N/A
		(c) Consider use of a flow diagram<br>**Comment**: N/A
Descriptive data	14*	(a) Give characteristics of study participants (eg demographic, clinical, social) and information on exposures and potential confounders<br>**Comment**: Demographic and clinical characteristics are clearly detailed in 3.1 and 3.2 chapters, as well as Table 1 and Figures 1 and 2. *(Results, Pages: 5-8, Lines: 216-318)*
		(b) Indicate number of participants with missing data for each variable of interest<br>**Comment**: N/A
Outcome data	15*	Report numbers in each exposure category, or summary measures of exposure<br>**Comment**: The exposures are diagnostic of RA (measured and classified according to current criteria) – 43 cases, COVID-19 (diagnosed through RT-PCR or Rapid-Antigen test) – 86 cases, and immunosuppressive treatments like DMARDs (32 cases used csDMARDs, 7 cases utilized tsDMARDs and in 19 cases we observed the use of a bDMARD). (*Materials and Methods, Page 4, Lines 159-186, Results, Page:14-15, Lines: 496-516)*
Main results	16	(*a*) Give unadjusted estimates and, if applicable, confounder-adjusted estimates and their precision (eg, 95% confidence interval). Make clear which confounders were adjusted for and why they were included<br>**Comment**: The results demonstrated that RA patients exhibit a distinct clinical and laboratory profile when compared to non-RA neurological patients with COVID-19. Severe COVID-19 outcomes were positively correlated with advanced age and specific laboratory markers, including ESR, leukocytes, neutrophils, neutrophil-to-lymphocyte ratio (NLR), AST, serum creatinine, and urea. SpO2 and lymphocyte levels were inversely correlated with COVID-19 severity. The analysis also highlighted a significant number of RA patients experiencing disease flares post-infection, necessitating modifications to their baseline treatments. *(Results, Pages: 6-16, Lines: 256-575)*
		(*b*) Report category boundaries when continuous variables were categorized<br>**Comment**: N/A
		(*c*) If relevant, consider translating estimates of relative risk into absolute risk for a meaningful time period<br>**Comment**: N/A
		Report other analyses done—eg analyses of subgroups and interactions, and sensitivity analyses<br>**Comment**: N/A
**Discussion**		
Key results	18	Summarise key results with reference to study objectives<br>**Comment**: The results aligned with the study's primary objective of investigating the clinical and paraclinical progression of COVID-19 in RA patients compared to non-RA patients. The study underscores the heightened risk of disease flares and severe outcomes in RA patients, particularly those receiving immunosuppressive therapy. *(Discussion, Pages: 17-19, Lines: 577-703)*
Limitations	19	Discuss limitations of the study, taking into account sources of potential bias or imprecision. Discuss both direction and magnitude of any potential bias<br>**Comment**: The limitations, including potential sources of bias are stated in the study. *(Discussions, Page: 19, Lines: 704-722)*

Interpretation	20	Give a cautious overall interpretation of results considering objectives, limitations, multiplicity of analyses, results from similar studies, and other relevant evidence<br>**Comment**: This issue was addressed in the "Discussion" Section. *(Discussion, Pages: 17-19, Lines: 577-722)*
Generalisability	21	Discuss the generalisability (external validity) of the study results<br>**Comment**: The findings contribute to the broader understanding of the interaction between autoimmune diseases like RA and COVID-19. However, due to the study's specific small sample size (Romanian RA patients), these findings should be cautiously generalized to other populations and settings. Future research involving larger, multicenter studies is necessary to validate these results and develop comprehensive management guidelines. *(Discussions, Page: 19, Lines: 712-722)*
**Other information**		
Funding	22	Give the source of funding and the role of the funders for the present study and, if applicable, for the original study on which the present article is based<br>**Comment**: N/A

Caption: **AST**: aspartate aminotransferase; **bDMARDs**: biological disease-modifying antirheumatic drugs; **COVID-19**: Coronavirus Disease 2019; **csDMARDs**: conventional synthetic disease-modifying antirheumatic drugs, **DMARD**: disease-modifying antirheumatic drugs, **ESR**: erythrocyte sedimentation rate**, NLR:** neutrophil-to-lymphocyte ratio, **SpO_2** - Peripheral capillary oxygen saturation; **tsDMARDs**: targeted-synthetic disease-modifying antirheumatic drugs, **RA**: rheumatoid arthritis; **RT-PCR** - Reverse transcription polymerase chain reaction
